# Burden and prevalence of risk factors for severe COVID-19 disease in the ageing European population - A SHARE-based analysis

**DOI:** 10.21203/rs.3.rs-73657/v1

**Published:** 2020-09-09

**Authors:** Linda Juel Ahrenfeldt, Camilla Riis Nielsen, Sören Möller, Kaare Christensen, Rune Lindahl-Jacobsen

**Affiliations:** University of Southern Denmark; University of Southern Denmark; Odense University Hospital; University of Southern Denmark; University of Southern Denmark

**Keywords:** COVID-19, SARS-CoV-2, prevalence, risk factors, burden of disease, Europe

## Abstract

**Aim:**

International health authorities suggest that individuals aged 65 years and above and people with underlying comorbidities such as hypertension, chronic lung disease, cardiovascular disease, cancer, diabetes, and obesity are at increased risk of severe Coronavirus Disease 2019 (COVID-19); however, the prevalence of risk factors is unknown in many countries. Therefore, we aim to describe the distribution of these risk factors across Europe.

**Subject and Methods:**

Prevalence of risk factors for severe COVID-19 was identified based on interview for 73,274 Europeans aged 50+ participating in the Survey of Health, Ageing and Retirement in Europe (SHARE) in 2017. Burden of disease was estimated using population data from Eurostat.

**Results:**

A total of 75.3% of the study population (corresponding to app. 60 million European men and 71 million women) had at least one risk factor for severe COVID-19,45.9% (app. 36 million men and 43 million women) had at least two factors and 21.2% (app. 17 million men and 20 million women) had at least three risk factors. The prevalences of underlying medical conditions ranged from 4.5% for cancer to 41.4% for hypertension, and the region-specific prevalence of having at least three risk factors ranged from 18.9% in Northern Europe to 24.6% in Eastern Europe.

**Conclusions:**

Information about the prevalences of risk factors might help authorities to identify the most vulnerable subpopulations with multiple risk factors of severe COVID-19 disease and thus to decide appropriate strategies to mitigate the pandemic.

## Introduction

During the last two decades, coronaviruses have caused major epidemics and outbreaks worldwide (Hon et al. 2020). In 2002, an epidemic of severe acute respiratory syndrome (SARS-CoV) was identified in China and spread subsequently to the rest of the world with a case-fatality of about 10% (Al-Hazmi 2016; Hon et al. 2020). In 2012, an epidemic of pneumonia occurred in Saudi Arabia, which was caused by another novel coronavirus called the Middle East respiratory syndrome (MERS-CoV) (Hon et al. 2020). MERS was identified in several countries in the gulf region, Korea and European region with a death rate of about 34% (Al-Hazmi 2016; Hon et al. 2020). In the winter 2019, a new outbreak of pneumonia occurred in Wuhan, China and spread rapidly over the world. A novel coronavirus (COVID-19) was identified as the underlying cause and has been named SARS-CoV-2 due to its similarities with SARS (Hon et al. 2020). These major outbreaks of coronavirus are similar in nature and in the mode of spread by droplets (Hon et al. 2020). A review of 25 papers found that older age, male sex and underlying medical conditions including diabetes mellitus, renal disease, respiratory disease, heart disease and hypertension were clinical predictors of death associated with MERS (Matsuyama et al. 2016). Among the five predictors, the largest risk of mortality was found for heart disease (odds ratio (OR) = 3.5) followed by respiratory disease (OR = 3.1). Similarly for SARS, older age and having multiple comorbidities such as diabetes mellitus, hypertension, and cerebrovascular disease were independent predictors of various adverse outcomes such as enrollment to an intensive care unit and death (Chan et al. 2003; Lien et al. 2008).

Deaths caused by COVID-19 have by August 20,2020 been confirmed to have passed more than 788,000 persons and 22 million persons are confirmed infected with the virus (JOHNS HOPKINS University & Medicine 2020). Guidelines from the Center for Disease Control and Prevention (CDC) (Centers for Disease Control and Prevention 2020) and European Centre for Disease Prevention and Control (ECDC) (European Centre for Disease Prevention and Control 2020) suggest that older people and those with underlying medical conditions such as hypertension, chronic lung disease, cardiovascular disease, cancer, diabetes, and obesity are at high risk of becoming severely ill (i.e. requiring hospitalization, intensive care, ventilator treatment to help breath) or dying from COVID-19.

The risk factors are primarily based on studies from Wuhan, China showing that the most severe and fatal cases of COVID-19 have occurred in elderly people and in patients with underlying comorbidities (Guan et al. 2020a; Guan et al. 2020b; Shahid et al. 2020; Singhal 2020; Wu and McGoogan 2020; Yang et al. 2020; Zhang et al. 2020; Zheng et al. 2020; Zhou et al. 2020). Based on the available evidence, age stands out as the predominant single risk factor for severe COVID-19 disease, but it is unknown, which of the underlying medical conditions are most important for developing severe disease (National Board of Health (Denmark) 2020; Norwegian Institute of Public Health 2020). However, a large cross-sectional study from the USA (Petrilli et al. 2020) describing characteristics of 4103 patients with COVID-19 disease, found that obesity (Body Mass Index (BMI) >30) was the chronic condition with the strongest association with critical illness (Petrilli et al. 2020). In line, a retrospective analysis (Lighter et al. 2020) found that among COVID-19 patients younger than 60 years, those with a BMI of 30–34 were twice as likely to be admitted to acute and critical care than patients with a BMI of less than 30. This was even more pronounced among patients with a BMI of 35 years and above (Lighter et al. 2020). Cardiovascular disease was a common comorbidity among people infected with SARS and MERS (Askin et al. 2020). A review of six studies including 1527 patients with COVID-19 (mainly from Wuhan) found that the most prevalent cardiovascular comorbidities were hypertension (17.1 %) and cardia-cerebrovascular disease (16.4%) followed by diabetes (9.7%) (Li et al. 2020). A systematic review and meta-analyses involving 23,736 cancer patients found that cancer patients with COVID-19 had higher mortality (OR = 2.54), however, largely driven by mortality among patients in China (Venkatesulu et al. 2020).

A recent study identified subpopulations at risk of severe COVID-19 using national health care and population registers on the total Swedish population (about 9 million individuals) with an average age of 41.4 years (Gémes et al. 2020). They found that 22.1 % of the population had at least one of six risk factors for severe COVID-19 (cardiovascular disease, cancer, chronic obstructive compulsory disease, severe asthma, diabetes and age 70+), 6.8% had at least two factors and 1.6% had at least three risk factors. They showed that the distribution of risk factors for severe COVID-19 differed across Sweden, and highlighted that the prevalence of risk factors is unknown in many countries (Gémes et al. 2020).

Here we examine the distribution of risk factors for severe COVID-19 in middle-aged and elderly men and women aged 50 and above from 26 European countries using data from the latest available wave of the Survey of Health, Ageing and Retirement in Europe (SHARE). We aim to estimate the prevalence (proportion) and burden (raw numbers) of risk factors to describe the distribution of these risk factors throughout Europe.

## Methods

### Setting and study population

SHARE is a large cross-national survey, collecting individual data about health, economic, and social factors of Europeans aged 50 and above. The SHARE data collection is done according to strict quality standards and with ex-ante harmonized interviews across the different countries (Borsch-Supan et al. 2013). To help reduce potential selection bias associated with nonresponse errors, SHARE provides calibrated weights. The calibrated cross-sectional weights in wave 7 are computed separately by country to match the size of national target populations of individuals age 50 and older in 2017 (SHARE 2020). To compensate for attrition, refresher respondents are added in each wave of SHARE (Borsch-Supan et al. 2013). The household response rate differ by wave and country and vary between 35.2% and 84.3% for refreshers in wave 7 (Bergmann et al. 2019).

We performed a large cross-sectional study including men and women aged 50 years and above from 26 European countries available in SHARE wave 7 (2017). We excluded individuals with unknown birth date(n = 26) and with missing cross-sectional individual weights (n = 173).

### Health variables

Based on guidelines from CDC (Centers for Disease Control and Prevention 2020) and ECDC (European Centre for Disease Prevention and Control 2020), the risk factors from SHARE selected for analyses were age 65 years and above, hypertension, chronic lung disease, cardiovascular disease, cancer, diabetes, and obesity (Table 1). Disease burden was based on the following interview question: “*Has a doctor ever told you that you had/do currently have any of the conditions on this card? [With this we mean that a doctor has told you that you have this condition, and that you are either currently being treated for or bothered by this condition]*”. The following diseases were included: “*High blood pressure or hypertension*”, “*Chronic lung disease such as chronic bronchitis or emphysema*”, “*Heart attack including myocardial infarction or coronary thrombosis or any other heart problem including congestive heart failure*”, “*A stroke or cerebral vascular disease*”, “*Cancer or malignant tumor, including leukemia or lymphoma, but excluding minor skin cancers*” and “*Diabetes or high blood sugar*”. Heart attack and stroke/ cerebrovascular disease were combined into one variable “Cardiovascular disease”. BMI was available based on self-reported height and weight with obesity defined as a BMI of 30 and above. Number of risk factors for severe COVID-19 was estimated based on the six underlying medical conditions (including obesity) and age 65+.

### Sociodemographic variables

Sociodemographic characteristics included age at interview, sex, European region and country. Age was divided into five intervals: 50–59,60–69,70–79,80–89 and 90+ years. In line with previous SHARE studies (Ahrenfeldt et al. 2019; Ahrenfeldt et al. 2018; Scheel-Hincke et al. 2019), the European countries were classified into four regions: Northern Europe (Denmark, Sweden and Finland), Western Europe (Austria, Germany, France, Switzerland, Belgium, and Luxembourg), Southern Europe (Spain, Italy, Greece, Portugal, Cyprus and Malta) and Eastern Europe (Czech Republic, Poland, Hungary, Slovenia, Estonia, Croatia, Lithuania, Bulgaria, Latvia, Romania, and Slovakia).

### Statistical analyses

We investigated the prevalence of risk factors for severe COVID-19 among individuals in SHARE wave 7. The prevalence of each medical condition and of having at least one, two and three risk factors were estimated in relation to European regions, age and sex, and the prevalence of people aged 65+ was estimated in relation to regions and sex. Furthermore, the prevalences of having at least one, two and three risk factors were mapped by country. As a sensitivity analysis, we investigated the prevalences of risk factors without including age as an underlying factor. All estimation of prevalences of risk factors included the individual cross-sectional probability weights given in the dataset (Borsch-Supan et al. 2013). Differences in prevalences between European regions, age and sex were investigated using logistic regressions. The burden for the risk factors in the total European population was estimated by multiplying the prevalence of risk factors in the SHARE population with the population number (assessed on January 1,2017) in the respective regions based on the Eurostat Database (Eurostat). Stata version 16.0 was used for all analyses. R version 3.6.3 was used to map the prevalences of risk factors.

## Results

The mean age in the study population was 68 years (age range 50–104 years). Among the 73,274 individuals, 43.3% were men and 56.7% were women. The study population was distributed with 11.0% in Northern Europe, 25.5% in Western Europe, 20.7% in Southern Europe and 42.4% in Eastern Europe (Supplementary Table 1). Among the underlying medical conditions less than one percent of data were missing except from obesity, where 3.7% were missing (not shown in table).

In total, 75.3% of the study population had at least one risk factor for severe COVID-19,45.9% had at least two factors and 21.2% had at least three factors. Overall, the prevalences for the risk factors were similar between European regions, except for higher prevalences in Eastern Europe (Table 2 and Supplementary Table 2). Prevalences of risk factors showed only minor differences for men across the European regions, whereas women in Eastern Europe had higher prevalences of risk factors compared with women in North, West and South (Table 2 and Supplementary Table 2). No sex differences were found in the overall prevalences of having at least one, two and three risk factors in Northern, Western and Southern Europe except for a lower prevalence of at least one risk factor for women than men (74.2% vs 76.1 %) in Southern Europe. In Eastern Europe, higher prevalences of having one, two and three risk factors were found for women than for men (79.6% vs 74.1 %, 53.5% vs 46.2% and 26.6% vs 22.1 %, respectively). Overall, more women than men were 65 years and above (53.8% vs 47.5%), and women had lower prevalences of lung disease (5.2% vs 6.1 %), cardiovascular disease (12.0% vs 16.0%) and diabetes (12.4% vs 14.4%) than men (Table 2 and Supplementary Table 3).

**Figure 1:**
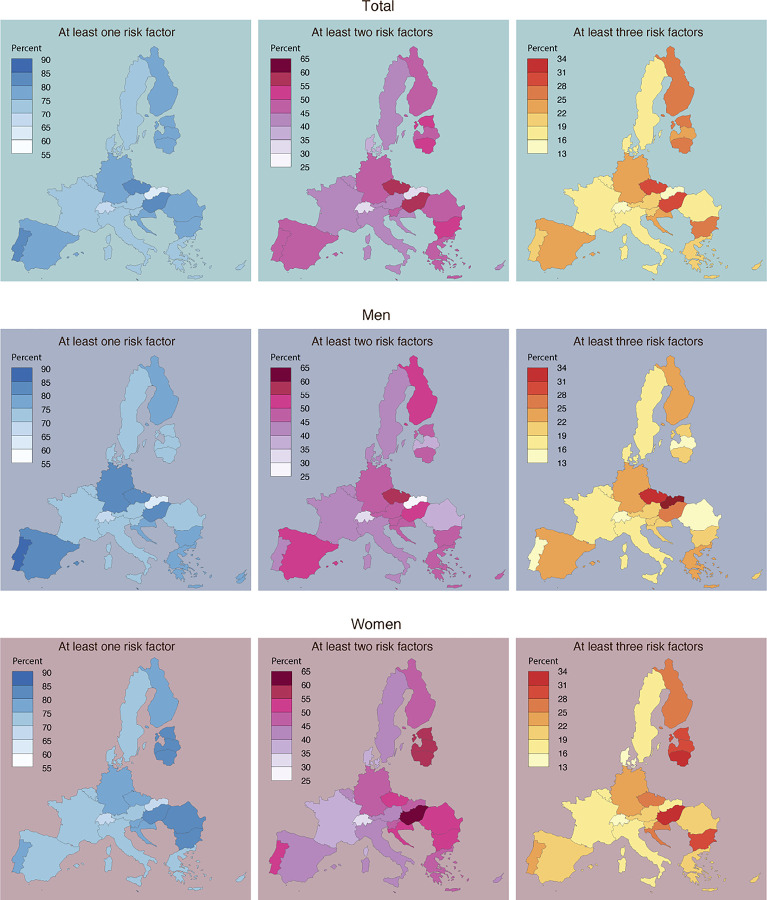
Maps demonstrating the prevalence of at least one, two and three risk factors for severe COVID-19 by European countries and sex.

When mapping the prevalences of risk factors in the 26 European countries (Figure 1), we found that the prevalence of having at least one risk factor ranged from 62.4% in Slovakia to 82.7% in Hungary; having at least two factors ranged from 33.5% in Slovakia to 57.7% in Hungary; and having at least three risk factors ranged from 13.5% in Slovakia to 29.2% in Czech Republic (Figure 1). For women, the highest prevalences of risk factors were found in Eastern Europe, with Hungary (83.5%), Lithuania (83.1 %) and Latvia (82.8%) having the highest prevalences of at least one risk factor, and Lithuania (59.0% and 31.1 %), Estonia (55.6% and 28.5%) and Latvia (55.0% and 30.1 %) having the highest prevalences of at least two and three risk factors, respectively. For men, a less consistent pattern was found. The highest prevalences of having at least one risk factor were found in Portugal (86.8%), Malta (86.6%) and Czech Republic (83.1 %), the highest prevalences of having at least two factors were found in Czech Republic (59.8%), Hungary (52.8%) and Malta (51.0%) followed by Spain (50.7%) and Finland (50.1 %), and the highest prevalences of having at least three factors were found in Czech Republic (31.8%), Hungary (26.0%) and Malta (25.4%) (Figure 1 and Supplementary Table 4).

The overall prevalence of the underlying medical conditions in the total sample was 41.4% for hypertension, 5.6% for chronic lung disease, 13.8% for cardiovascular disease, 4.5% for cancer, 13.3% for diabetes and 21.4% for obesity, but varied between European regions (Table 2). The most pronounced differences between regions were found for Eastern Europe. Compared with Northern and Western Europe, Eastern Europe had higher prevalences of hypertension, cardiovascular disease, diabetes and obesity, but lower prevalences of lung disease and cancer. Compared with Southern Europe, Eastern Europe had higher prevalences of hypertension, cardiovascular disease and obesity (Table 2 and Supplementary Table 2).

Overall, the prevalences of having at least one, two and three risk factors increased with age until the age group 80–89 years but decreased thereafter. Also, the prevalence of each medical condition generally increased with age; however, the prevalences of obesity increased until age 60–69 years, were overall stable until age 80–89 years, and decreased thereafter (Table 2 and Supplementary Table 5).

When estimating the prevalences of risk factors without age as an underlying factor, the overall prevalences of having at least one, two and three risk factors decreased to 60.3%, 27.9% and 9.5%, respectively (Supplementary Table 6).

Given the prevalences found in the SHARE population, we estimated that the number of individuals having at least one of the risk factors for severe COVID-19 in the European population was about 131 million persons (70,672,673 women and 60,104,205 men). Almost 80 million persons (42,592,064 women and 36,253,330 men) had at least two risk factors and almost 37 million persons (20,165,269 women and 16,695,613 men) had at least three risk factors (Table 3). The burden of the underlying medical conditions ranged from about 8 million persons with cancer to about 72 million persons with hypertension. The region-specific burden of having at least three risk factors ranged from 1.5 million persons in Northern Europe to 9.5 million persons in Eastern Europe (Table 3). The burden of risk factors in the specific age groups can be found in Supplementary Table 7.

## Discussion

Using data from SHARE wave 7, we found that the main part of the study population aged 50+ years (about 75% corresponding to app. 60 million European men and 71 million women) had at least one risk factor for severe COVID-19,46% (app. 36 million men and 43 million women) had at least two factors and 21 % (app. 17 million men and 20 million women) had at least three risk factors. The prevalence of underlying medical conditions ranged from 4.5% for cancer to 41.4% for hypertension. Overall, the prevalences of risk factors increased with age until 80–89 years. Generally, the prevalences were similar between the European regions, except for higher prevalences of risk factors in Eastern Europe, mainly explained by a higher prevalence for women in Eastern Europe compared with women in Northern, Western and Southern Europe.

Compared with the recent study identifying subpopulations at risk of severe COVID-19 in Sweden (Gémes et al. 2020), we found higher prevalences of risk factors. This is at least partly explained by the differences in average age in the two study populations (41 vs 68 years) and the different ways information was gathered. The Swedish data was based on hospitalizations, representing those with most severe disease, with diagnoses of medical conditions within three years prior to January 1,2016 to capture individuals with active disease. Our study was based on self-reported conditions. Thus, we capture both individuals with active disease, but also persons who had the specific disease back in time. In line, the Swedish study found an overall greater burden of risk factors as the look back period increased (Gémes et al. 2020).

In line with the regional differences observed in this study, other SHARE-studies demonstrated the highest prevalence of disability in Eastern and Southern Europe (Scheel-Hincke et al. 2019) and the lowest physical strength in Eastern Europe (Ahrenfeldt et al. 2018). Another recent study found that Eastern Europe had the highest proportion of individuals with a comorbidity index of two or more, and that women had more comorbidity than men in Eastern, Southern, and Northern Europe, but most pronounced in Eastern Europe (Ahrenfeldt et al. 2019).

In agreement with observations in this study showing overall similar prevalences of risk factors between men and women, available sex-disaggregated data for COVID-19 show equal numbers of cases between sexes (Spagnolo et al. 2020). However, men with COVID-19 are more at risk for worse outcomes and death, independent of age (Jin et al. 2020). Plausible reasons for this sex difference include high risk behaviors, immune response, biological differences between the sexes and the contribution of underlying cardiovascular risk factors, which is higher among men than among women (Sharma et al. 2020).

To prevent severe disease of COVID-19, it is important for national and international authorities and local governments to identify the possible subpopulations with increased risk. Current evidence on COVID-19 from China found that the hazard ratio of reaching the composite endpoint (i.e. admission to an intensive care unit, invasive ventilation or death) was 1.79 for patients with at least one comorbidity and 2.59 for patients with two or more comorbidities (Guan et al. 2020a). In agreement with global estimates (Clark et al. 2020), results from the present study show that large parts of the middle-aged and elderly populations will have at least one risk factor for severe COVID-19 making interventions towards all persons with risk factors infeasible; nevertheless, identifying the most vulnerable subpopulations with multiple risk factors, shown here to be about one fifth of the European population, may help authorities in planning healthcare resources and to decide appropriate strategies to mitigate the pandemic and reduce mortality from the disease (Gémes et al. 2020).

The main strength of this study is the large national samples of participants from 26 European countries with harmonized disease measures (Borsch-Supan et al. 2013), making it possible to visualize the prevalence and burden of risk factors for severe COVID-19 by European regions and countries. Although we used the latest wave of SHARE, the present study was based on data from 2017. Thus, if the structure of the European population has changed during the last three years, the prevalence estimates may be different now. The medical conditions included in the analysis are all some of the most prevalent underlying conditions observed among COVID-19 cases with severe disease according to ECDC (European Centre for Disease Prevention and Control 2020) and CDC (Centers for Disease Control and Prevention 2020). However, still a lot is unknown about the number and the impact of risk factors on COVID-19 and there might be more relevant risk factors than the ones considered here. While the World Health Organization (WHO) also list a number of underlying medical conditions associated with high risk of developing serious COVID-19 illness, they do not define what “serious illness” entails, and thus we have not referred to WHO in this paper. Nevertheless, many of the risk factors mentioned by WHO is the same as mentioned by ECDC and CDC (World Health Organization 2020). Limitations to the SHARE data are that the sampling procedures might vary by country and the low response rate in some countries. However, to try to correct for potential selection into the study population, SHARE provides calibrated probability weights, which are constructed to reduce the impact of both unit and item nonresponse (Borsch-Supan et al. 2013; SHARE 2020). Moreover, misclassification is an issue because diseases are self-reported; however, by using self-reported data in contrast to register-based hospitalization data, we were able to calculate the prevalences of risk factors of COVID-19 among the general European population, and not only among people with most severe disease requiring hospital admission.

This is to our knowledge the largest study to date based on individual level data describing the prevalence and burden of risk factors for COVID-19 across European regions. About 75% of the study population (corresponding to app. 60 million European men and 71 million women) had at least one risk factor for COVID-19, whereas about 21 % (app. 17 million men and 20 million women) had at least three risk factors. The prevalence of the underlying medical conditions ranged from 4.5% for cancer to 41.4% for hypertension. Generally, the prevalences were similar between the European regions, except for higher prevalences of risk factors in Eastern Europe particularly among women. Information about the prevalences of risk factors might help authorities to identify the most vulnerable subpopulations with multiple risk factors of severe COVID-19 disease and thus to decide appropriate strategies to mitigate the pandemic.

## Supplementary Material

Supplement

## Figures and Tables

**Table 1 -:** Identified risk factors for severe COVID-19 by the Center for Disease Control and Prevention (CDC) and European Centre for Disease Prevention and Control (ECDC), and data selected for the present study from the Survey of Health, Ageing and Retirement in Europe (SHARE)

CDC^a^	ECDC^b^	Risk factors from SHARE selected for the present study
65 years and above	70 years and above	65 years and above
	Hypertension	High blood pressure or hypertension
People with diabetes	Diabetes	Diabetes or high blood sugar
People who have serious heart conditions	Cardiovascular Diseases	Cardiovascular Disease (Heart attack including myocardial infarction or coronary thrombosis or any other heart problem including congestive heart failure and stroke or cerebral vascular disease)
People with chronic lung disease or moderate to severe asthma	Chronic Respiratory Diseases	Chronic lung disease such as chronic bronchitis or emphysema
	Cancer	Cancer or malignant tumor including leukemia or lymphoma excluding minor skin cancers
People with severe obesity (BMI 40+)	Obesity (BMI 30–40+)	BMI = 30+
People with liver disease		
People with chronic kidney disease undergoing dialysis		
People who live in a nursing home or long-term care facility		
People who are immunocompromised (cancer treatment, smoking, bone marrow or organ transplantation, immune deficiencies, poorly controlled HIV or AIDS, and prolonged use of corticosteroids and other immune weakening medications)	Immune compromised status	

Accessed August 20,2020

a Centers for Disease Control and Prevention. Coronavirus Disease 2019 (COVID-19). https://www.cdc.gov/coronavirus/2019-ncov/need-extra-precautions/people-at-higher-risk.html

b European Centre for Disease Prevention and Control. Disease background of COVID-19.2020. https://www.ecdc.europa.eu/en/2019-ncov-background-disease

**Table 2 -:** Prevalence of risk factors for COVID-19 by European region and age group for the total population and stratified by sex

	Age 65 and above	Hypertension	Chronic lung disease	Cardiovascular disease	Cancer	Diabetes	Obesity	At least one prognos-tic factor	At least two prognos-tic factors	At least three prognos-tic factors
**All countries**										
Total	51.0	41.4	5.6	13.8	4.5	13.3	21.4	75.3	45.9	21.2
Men	47.7 ^a^	40.8	6.1 ^a^	16.0 ^a^	4.6	14.4 ^a^	21.5	75.6	45.6	21.0
Women	53.8	41.9	5.2	12.0	4.5	12.4	21.4	75.0	45.2	21.4
**Northern EU**										
Total	52.5	38.1	6.0	12.5	5.6	11.1	19.5	74.3	43.3	18.9
Men	50.5 ^a^	38.6	5.6	14.5 ^a^	5.5	13.5 ^a^	19.2	74.9	44.7	19.0
Women	54.3	37.6	6.3	10.7	5.6	8.9	19.7	73.7	42.1	18.9
**Western EU**										
Total	50.6	39.0	6.6	14.1	5.8	12.3	21.8	75.1	45.1	20.3
Men	47.4 ^a^	39.8	6.6	17.1 ^a^	5.9	14.3 ^a^	22.7	76.2	45.9	21.1
Women	53.4	38.2	6.6	11.5	5.6	10.6	21.1	74.2	44.4	19.7
**Southern EU**										
Total	52.8	41.6	4.9	11.8	3.3	14.6	18.9	74.2	44.3	20.2
Men	50.1 ^a^	42.8	6.5 ^a^	13.9 ^a^	3.3	15.8 ^a^	21.8	76.1 ^a^	45.0	20.5
Women	55.1	40.6	3.5	10.1	3.4	13.7	17.3	72.7	43.7	20.0
**Eastern EU**										
Total	49.0	47.7	4.9	16.4	3.8	14.3	29.1	77.2	50.3	24.6
Men	44.3 ^a^	42.0 ^a^	5.1	17.2	3.7	13.5	18.2	74.1 ^a^	46.2 ^a^	22.1 ^a^
Women	52.6	52.2	4.7	15.9	3.9	14.8	27.8	79.6	53.5	26.6
**Age 50–59**										
Total	-	28.8	4.3	6.4	3.1	8.1	21.6	46.7	17.7	5.7
Men	-	32.0 ^a^	5.3 ^a^	8.9 ^a^	2.5	9.6 ^a^	22.8	51.5 ^a^	20.1 ^a^	7.0 ^a^
Women	-	25.7	3.3	4.0	3.6	6.6	20.5	42.1	15.4	4.4
**Age 60–69**										
Total	-	41.6	5.2	11.4	4.4	13.0	25.0	77.2	44.2	19.3
Men	-	42.1	5.1	14.5 ^a^	4.0	14.9 ^a^	25.0	78.3 ^a^	46.1 ^a^	20.5 ^a^
Women	-	41.1	5.3	8.6	4.8	11.3	25.0	76.2	42.5	18.2
**Age 70–79**										
Total	-	53.0	6.8	19.5	6.4	18.5	21.2	100	71.8	36.0
Men	-	50.3 ^a^	7.2	22.9 ^a^	7.7 ^a^	20.6 ^a^	18.9 ^a^	100	72.9	36.9
Women	-	55.1	6.5	16.9	5.4	16.9	23.0	100	70.9	35.3
**Age 80–89**										
Total	-	56.1	8.2	27.2	5.6	19.2	19.8	100	77.2	40.1
Men	-	51.8 ^a^	10.2 ^a^	30.0 ^a^	7.7 ^a^	18.5	17.1 ^a^	100	76.8	39.2
Women	-	58.6	6.9	25.5	4.4	19.6	21.4	100	77.4	40.6
**Age 90+**										
Total	-	47.4	7.5	28.5	5.7	17.3	8.7	100	68.2	31.7
Men	-	41.4	10.0	27.2	5.2	17.6	5.9	100	63.4	29.1
Women	-	49.9	6.5	29.0	6.0	17.1	10.0	100	70.2	32.8

a Significant sex difference (p<0.05)

Northern Europe: Sweden, Denmark and Finland

Western Europe: Austria, Germany, France, Switzerland, Belgium and Luxembourg

Southern Europe: Spain, Italy, Greece, Portugal, Malta and Cyprus

Eastern Europe: Czech Republic, Poland, Hungary, Slovenia, Estonia, Croatia, Lithuania, Bulgaria, Latvia, Romania and Slovakia

**Table 3-:** Burden (raw numbers) of risk factors for Covid-19 by European regions

	Age 65+	Hypertension	Chronic lung disease	Cardiovascular disease	Cancer	Diabetes	Obesity	At least one risk factor	At least two risk factors	At least three risk factors
**All countries**										
Total	85,293,598	71,925,523	9,729,056	23,975,174	7,817,992	23,106,509	37,178,894	130,821,060	79,743,515	36,831,427
Men	36,259,364	32,437,190	4,849,678	12,720,467	3,657,134	11,448,420	17,093,127	60,104,205	36,253,330	16,695,613
Women	49,034,234	39,482,467	4,899,972	11,307,628	4,240,360	11,684,549	20,165,269	70,672,673	42,592,064	20,165,269
**Northern EU**										
Total	4,222,004	3,148,568	495,837	1,032,995	462,782	917,299	1,611,472	6,140,120	3,578,293	1,561,888
Men	1,914,850	1,520,280	220,559	571,090	216,620	531,704	756,201	2,949,973	1,760,531	748,324
Women	2,307,154	1,626,353	272,501	462,819	242,223	384,961	852,105	3,187,826	1,820,997	817,502
**Western EU**										
Total	18,278,979	28,474,960	4,818,839	10,294,793	4,234,738	8,980,564	15,916,773	54,832,552	32,928,736	14,821,582
Men	7,189,170	13,474,951	2,234,540	5,789,489	1,997,543	4,841,503	7,685,462	25,798,776	15,540,207	7,143,755
Women	11,089,809	14,957,614	2,584,300	4,502,947	2,192,739	4,150,542	8,261,928	29,053,795	17,385,290	7,713,743
**Southern EU**										
Total	27,067,173	22,305,719	2,627,356	6,327, 103	1,769,444	7,828,449	9,758,752	39,785,682	23,753,446	10,831,142
Men	11,697,857	10,539,366	1,600,605	3,422,831	812,615	3,890,701	4,260,071	18,739,387	11,081,109	5,048,061
Women	15,369,316	11,771,901	1,014,819	2,928,478	985,824	3,972,292	5,480,023	21,079,241	12,670,741	5,798,966
**Eastern EU**										
Total	35,725,442	18,525,228	1,903,011	6,369,261	1,475,804	5,553,685	11,107,369	29,982,129	19,534,988	9,553,891
Men	15,457,487	7,174,868	871,234	2,938,279	632,072	2,306,208	4,749,080	12,658,518	7,892,355	3,775,347
Women	20,267,955	11,355,554	1,022,435	3,458,876	848,403	3,219,582	6,330,395	17,316,132	11,638,355	5,786,547

Data was estimated by multiplying the prevalence of risk factors in the SHARE population (Table 2) with the population number (assessed on January 1, 2017) in the respective countries based on the Eurostat Database

Northern Europe: Sweden, Denmark and Finland

Western Europe: Austria, Germany, France, Switzerland, Belgium and Luxembourg

Southern Europe: Spain, Italy, Greece, Portugal, Malta and Cyprus

Eastern Europe: Czech Republic, Poland, Hungary, Slovenia, Estonia, Croatia, Lithuania, Bulgaria, Latvia, Romania and Slovakia
