# Supplementary material for: Burden and prevalence of risk factors for severe COVID-19 disease in the ageing European population - A SHARE-based analysis
Source: Res Sq. 2020 Sep 9:rs.3.rs-73657. Preprint. [Version 1] doi: 10.21203/rs.3.rs-73657/v1 (PMC7491580; doi:10.21203/rs.3.rs-73657/v1)
Supplement: Supplement [file SuppTables.docx]

**Supplementary Tables**

Supplementary Table 1 - Characteristics of the study population (The Survey of Health, Ageing and Retirement in Europe (SHARE) wave 7, 2017)

|  | Men | Women | Total |
| --- | --- | --- | --- |
| **Numbers (%)** | 31,751 (43.3) | 41,523 (56.7) | 73,274 (100.0) |
| **Age groups** |  |  |  |
| 50-59 | 6,315 (19.9) | 9,253 (22.3) | 15,568 (21.3) |
| 60-69 | 12,115 (38.2) | 14,754 (35.5) | 26,869 (36.7) |
| 70-79 | 8,969 (28.3) | 11,170 (26.9) | 20,139 (27.5) |
| 80-89 | 3,905 (12.3) | 5,488 (13.2) | 9,393 (12.8) |
| 90+ | 447 (1.4) | 858 (2.1) | 1,305 (1.8) |
| **Proxy interview** |  |  |  |
| Respondent only | 29,994 (94.5) | 39,903 (96.1) | 69,897 (95.4) |
| Respondent and proxy | 658 (2.1) | 616 (1.5) | 1,274 (1.7) |
| Proxy only | 999 (3.2) | 902 (2.2) | 1,901 (2.6) |
| Missing | 100 (0.3) | 102 (0.3) | 202 (0.3) |
| **Region and countries** |  |  |  |
| **Northern EU** | 3,858 (12.2) | 4,495 (10.8) | 8,353 (11.4) |
| Denmark | 1,472 (4.6) | 1,730 (4.2) | 3,202 (4.4) |
| Sweden | 1,469 (4.6) | 1,711 (4.1) | 3,180 (4.3) |
| Finland | 917 (2.9) | 1,054 (2.5) | 1,971 (2.7) |
| **Western EU** | 8,279 (26.1) | 10,418 (25.1) | 18,697 (25.5) |
| Austria | 1,298 (4.1) | 1,873 (4.5) | 3,171 (4.3) |
| Germany | 1,785 (5.2) | 2,002 (4.8) | 3,788 (5.2) |
| France | 1,384 (4.4) | 1,902 (4.6) | 3,286 (4.5) |
| Switzerland | 1,082 (3.4) | 1,300 (3.1) | 2,382 (3.3) |
| Belgium | 2,162 (6.8) | 2,673 (6.4) | 4,835 (6.6) |
| Luxembourg | 568 (1.8) | 670 (1.6) | 1,238 (1.7) |
| **Southern EU** | 6,678 (21.0) | 8,472 (20.4) | 15,150 (20.7) |
| Spain | 2,071 (6.5) | 2,599 (6.3) | 4,670 (6.4) |
| Italy | 2,047 (6.5) | 2,462 (5.9) | 4,509 (6.2) |
| Greece | 1,296 (4.1) | 1,736 (4.2) | 3,032 (4.1) |
| Portugal | 222 (0.7) | 282 (0.7) | 504 (0.7) |
| Cyprus | 492 (1.6) | 703 (1.7) | 1,195 (1.6) |
| Maltha | 550 (1.7) | 690 (1.7) | 1,240 (1.7) |
| **Eastern EU** | 12,936 (40.7) | 18,138 (43.7) | 31,074 (42.4) |
| Czech Republic | 1,677 (5.3) | 2,508 (6.0) | 4,185 (5.7) |
| Poland | 2,080 (6.6) | 2,545 (6.1) | 4,625 (6.3) |
| Hungary | 607 (1.9) | 921 (2.2) | 1,528 (2.1) |
| Slovenia | 1,546 (4.9) | 2,129 (5.1) | 3,675 (5.0) |
| Estonia | 1,963 (6.2) | 3,096 (7.5) | 5,059 (6.9) |
| Croatia | 1,059 (3.3) | 1,312 (3.2) | 2,371 (3.2) |
| Lithuania | 714 (2.3) | 1,261 (3.0) | 1,975 (2.7) |
| Bulgaria | 835 (2.6) | 1,109 (2.7) | 1,944 (2.7) |
| Latvia | 626 (2.0) | 1,079 (2.6) | 1,705 (2.3) |
| Romania | 892 (2.8) | 1,143 (2.8) | 2,035 (2.8) |
| Slovakia | 937 (3.0) | 1,035 (2.5) | 1,972 (2.7) |

All data are N (%)

Supplementary Table 2 - P-values for differences between European regions regarding risk factors for COVID-19 for the total study population and stratified for men and women

|  | Age 65+ | Hypertension | Lung disease | Cardiovascular disease | Cancer | Diabetes | Obesity | At least one prognos-tic factor | At least two prognos-tic factors | At least three prognos-tic factors |
| --- | --- | --- | --- | --- | --- | --- | --- | --- | --- | --- |
| **Northern Europe (ref)** |  |  |  |  |  |  |  |  |  |  |
| Western Europe | 0.064 | 0.365 | 0.169 | 0.017 | 0.690 | 0.058 | 0.006 | 0.367 | 0.075 | 0.063 |
| Southern Europe | 0.815 | 0.001 | 0.103 | 0.308 | <0.001 | <0.001 | 0.157 | 0.990 | 0.376 | 0.112 |
| Eastern Europe | <0.001 | <0.001 | 0.005 | <0.001 | <0.001 | <0.001 | <0.001 | 0.001 | <0.001 | <0.001 |
| **Western Europe (ref)** |  |  |  |  |  |  |  |  |  |  |
| Northern Europe | 0.064 | 0.365 | 0.169 | 0.017 | 0.690 | 0.058 | 0.006 | 0.367 | 0.075 | 0.063 |
| Southern Europe | 0.037 | 0.006 | 0.008 | <0.001 | <0.001 | <0.001 | <0.001 | 0.370 | 0.430 | 0.864 |
| Eastern | 0.036 | <0.001 | <0.001 | <0.001 | <0.001 | <0.001 | <0.001 | 0.005 | <0.001 | <0.001 |
| **Southern Europe (ref)** |  |  |  |  |  |  |  |  |  |  |
| Northern Europe | 0.815 | 0.001 | 0.103 | 0.308 | <0.001 | <0.001 | 0.157 | 0.990 | 0.376 | 0.112 |
| Western Europe | 0.037 | 0.006 | 0.008 | <0.001 | <0.001 | <0.001 | <0.001 | 0.370 | 0.430 | 0.864 |
| Eastern Europe | <0.001 | <0.001 | 0.933 | <0.001 | 0.137 | 0.562 | <0.001 | 0.001 | <0.001 | <0.001 |
| **Eastern Europe (ref)** |  |  |  |  |  |  |  |  |  |  |
| Northern Europe | <0.001 | <0.001 | 0.005 | <0.001 | <0.001 | <0.001 | <0.001 | 0.001 | <0.001 | <0.001 |
| Western Europe | 0.036 | <0.001 | <0.001 | <0.001 | <0.001 | <0.001 | <0.001 | 0.005 | <0.001 | <0.001 |
| Southern Europe | <0.001 | <0.001 | 0.933 | <0.001 | 0.137 | 0.562 | <0.001 | 0.001 | <0.001 | <0.001 |
| **Men** |  |  |  |  |  |  |  |  |  |  |
| **Northern Europe (ref)** |  |  |  |  |  |  |  |  |  |  |
| Western Europe | 0.039 | 0.398 | 0.128 | 0.010 | 0.516 | 0.425 | 0.008 | 0.387 | 0.408 | 0.049 |
| Southern Europe | 0.808 | 0.013 | 0.366 | 0.550 | 0.001 | 0.052 | 0.162 | 0.518 | 0.833 | 0.212 |
| Eastern Europe | <0.001 | 0.016 | 0.404 | 0.003 | <0.001 | 0.955 | <0.001 | 0.560 | 0.291 | 0.003 |
| **Western Europe (ref)** |  |  |  |  |  |  |  |  |  |  |
| Northern Europe | 0.039 | 0.398 | 0.128 | 0.010 | 0.516 | 0.425 | 0.008 | 0.387 | 0.408 | 0.049 |
| Southern Europe | 0.087 | 0.053 | 0.963 | 0.002 | <0.001 | 0.159 | <0.001 | 0.916 | 0.573 | 0.548 |
| Eastern Europe | 0.008 | 0.073 | 0.004 | 0.886 | <0.001 | 0.349 | <0.001 | 0.065 | 0.827 | 0.328 |
| **Southern Europe (ref)** |  |  |  |  |  |  |  |  |  |  |
| Northern Europe | 0.808 | 0.013 | 0.366 | 0.550 | 0.001 | 0.052 | 0.162 | 0.518 | 0.833 | 0.212 |
| Western Europe | 0.087 | 0.053 | 0.963 | 0.002 | <0.001 | 0.159 | <0.001 | 0.916 | 0.573 | 0.548 |
| Eastern Europe | <0.001 | 0.570 | 0.136 | <0.001 | 0.470 | 0.023 | <0.001 | 0.182 | 0.441 | 0.137 |
| **Eastern Europe (ref)** |  |  |  |  |  |  |  |  |  |  |
| Northern Europe | <0.001 | 0.016 | 0.404 | 0.003 | <0.001 | 0.955 | <0.001 | 0.560 | 0.291 | 0.003 |
| Western Europe | 0.008 | 0.073 | 0.004 | 0.886 | <0.001 | 0.349 | <0.001 | 0.065 | 0.827 | 0.328 |
| Southern Europe | <0.001 | 0.570 | 0.136 | <0.001 | 0.470 | 0.023 | <0.001 | 0.182 | 0.441 | 0.137 |
| **Women** |  |  |  |  |  |  |  |  |  |  |
| **Northern Europe (ref)** |  |  |  |  |  |  |  |  |  |  |
| Western Europe | 0.509 | 0.646 | 0.652 | 0.380 | 0.982 | 0.034 | 0.214 | 0.665 | 0.084 | 0.457 |
| Southern Europe | 0.622 | 0.037 | <0.001 | 0.479 | <0.001 | <0.001 | 0.536 | 0.526 | 0.271 | 0.309 |
| Eastern Europe | 0.191 | <0.001 | 0.003 | <0.001 | 0.002 | <0.001 | <0.001 | <0.001 | <0.001 | <0.001 |
| **Western Europe (ref)** |  |  |  |  |  |  |  |  |  |  |
| Northern Europe | 0.509 | 0.646 | 0.652 | 0.380 | 0.982 | 0.034 | 0.214 | 0.665 | 0.084 | 0.457 |
| Southern Europe | 0.219 | 0.054 | <0.001 | 0.058 | <0.001 | <0.001 | 0.044 | 0.252 | 0.588 | 0.715 |
| Eastern Europe | 0.456 | <0.001 | <0.001 | <0.001 | <0.001 | <0.001 | <0.001 | <0.001 | <0.001 | <0.001 |
| **Southern Europe (ref)** |  |  |  |  |  |  |  |  |  |  |
| Northern Europe | 0.622 | 0.037 | <0.001 | 0.479 | <0.001 | <0.001 | 0.536 | 0.526 | 0.271 | 0.309 |
| Western Europe | 0.219 | 0.054 | <0.001 | 0.058 | <0.001 | <0.001 | 0.044 | 0.252 | 0.588 | 0.715 |
| Eastern Europe | 0.055 | <0.001 | 0.013 | <0.001 | 0.175 | 0.153 | <0.001 | <0.001 | <0.001 | <0.001 |
| **Eastern Europe (ref)** |  |  |  |  |  |  |  |  |  |  |
| Northern Europe | 0.191 | <0.001 | 0.003 | <0.001 | 0.002 | <0.001 | <0.001 | <0.001 | <0.001 | <0.001 |
| Western Europe | 0.456 | <0.001 | <0.001 | <0.001 | <0.001 | <0.001 | <0.001 | <0.001 | <0.001 | <0.001 |
| Southern Europe | 0.055 | <0.001 | 0.013 | <0.001 | 0.175 | 0.153 | <0.001 | <0.001 | <0.001 | <0.001 |

Supplementary Table 3 - P-values for sex differences regarding risk factors for COVID-19

|  | Age 65+ | Hypertension | Lung disease | Cardiovascular disease | Cancer | Diabetes | Obesity | At least one risk factor | At least two risk factors | At least three risk factors |
| --- | --- | --- | --- | --- | --- | --- | --- | --- | --- | --- |
| All | <0.001 | 0.162 | 0.019 | <0.001 | 0.769 | <0.001 | 0.661 | 0.354 | 0.440 | 0.570 |
| Northern | 0.020 | 0.545 | 0.262 | <0.001 | 0.909 | <0.001 | 0.741 | 0.420 | 0.107 | 0.886 |
| Western | <0.001 | 0.179 | 0.942 | <0.001 | 0.530 | <0.001 | 0.120 | 0.079 | 0.211 | 0.107 |
| Southern | 0.003 | 0.167 | 0.001 | <0.001 | 0.913 | 0.044 | 0.202 | 0.043 | 0.406 | 0.653 |
| Eastern | <0.001 | <0.001 | 0.315 | 0.065 | 0.416 | 0.081 | 0.180 | <0.001 | <0.001 | <0.001 |
| age 50-59 |  | <0.001 | 0.020 | <0.001 | 0.100 | 0.002 | 0.087 | <0.001 | <0.001 | 0.001 |
| age 60-69 |  | 0.336 | 0.626 | <0.001 | 0.054 | <0.001 | 0.959 | 0.026 | 0.001 | 0.005 |
| age 70-79 |  | <0.001 | 0.272 | <0.001 | <0.001 | <0.001 | <0.001 |  | 0.074 | 0.179 |
| age 80-89 |  | <0.001 | 0.001 | 0.005 | <0.001 | 0.437 | 0.006 |  | 0.696 | 0.442 |
| age 90+ |  | 0.075 | 0.189 | 0.676 | 0.721 | 0.891 | 0.115 |  | 0.157 | 0.402 |

Supplementary Table 4 - Prevalence (in percent) of risk factors for COVID-19 by European countries

|  | At least one risk factor | | | At least two risk factors | | | At least three risk factors | | |
| --- | --- | --- | --- | --- | --- | --- | --- | --- | --- |
|  | Total | Men | Women | Total | Men | Women | Total | Men | Women |
| Denmark | 72.3 | 72.0 | 72.6 | 39.5 | 41.7 | 37.4 | 16.3 | 17.6 | 15.0 |
| Sweden | 73.7 | 74.1 | 73.4 | 41.9 | 43.3 | 40.6 | 16.6 | 17.0 | 16.2 |
| Finland | 77.1 | 79.4 | 75.1 | 49.5 | 50.1 | 49.0 | 25.4 | 23.9 | 26.7 |
| Austria | 73.5 | 73.5 | 73.4 | 44.4 | 45.1 | 43.8 | 19.8 | 19.3 | 20.2 |
| Germany | 78.9 | 80.9 | 77.1 | 49.8 | 49.7 | 49.8 | 23.7 | 24.5 | 22.9 |
| France | 71.4 | 71.5 | 71.3 | 40.9 | 42.9 | 39.1 | 17.3 | 18.3 | 16.6 |
| Switzerland | 66.0 | 66.2 | 65.9 | 32.1 | 33.9 | 30.5 | 12.1 | 12.8 | 11.4 |
| Belgium | 74.7 | 74.6 | 74.7 | 42.1 | 41.6 | 42.5 | 17.4 | 18.1 | 16.8 |
| Luxembourg | 72.9 | 73.7 | 72.2 | 39.5 | 41.5 | 37.5 | 18.3 | 20.6 | 16.1 |
| Spain | 77.0 | 80.0 | 74.5 | 47.0 | 50.7 | 43.8 | 22.9 | 24.5 | 21.6 |
| Italy | 71.2 | 71.4 | 71.0 | 41.4 | 41.1 | 41.6 | 18.2 | 18.2 | 18.3 |
| Greece | 74.3 | 77.0 | 72.0 | 48.7 | 48.1 | 49.2 | 21.7 | 22.9 | 20.7 |
| Portugal | 81.0 | 86.8 | 76.3 | 46.5 | 41.6 | 50.3 | 19.3 | 15.1 | 22.7 |
| Cyprus | 74.6 | 76.9 | 72.6 | 44.9 | 46.4 | 43.6 | 21.1 | 20.7 | 21.4 |
| Malta | 81.5 | 86.6 | 80.5 | 50.3 | 51.0 | 49.7 | 22.2 | 25.4 | 19.3 |
| Czech Republic | 81.2 | 83.1 | 79.6 | 56.9 | 59.8 | 54.5 | 29.2 | 31.8 | 27.0 |
| Poland | 75.9 | 72.6 | 78.5 | 49.9 | 46.3 | 52.7 | 26.4 | 23.9 | 28.3 |
| Hungary | 82.7 | 81.7 | 83.5 | 57.7 | 52.8 | 61.4 | 29.8 | 26.0 | 32.7 |
| Slovenia | 73.7 | 71.8 | 75.3 | 46.2 | 46.7 | 45.7 | 21.3 | 21.2 | 21.4 |
| Estonia | 77.9 | 73.8 | 80.6 | 51.7 | 45.9 | 55.6 | 25.0 | 19.8 | 28.5 |
| Croatia | 77.4 | 77.0 | 77.8 | 41.5 | 48.2 | 54.1 | 24.0 | 21.7 | 25.8 |
| Lithuania | 79.4 | 73.6 | 83.1 | 53.9 | 46.2 | 59.0 | 27.2 | 21.0 | 31.3 |
| Bulgaria | 79.3 | 77.8 | 80.4 | 51.2 | 46.7 | 54.8 | 25.3 | 21.8 | 28.1 |
| Latvia | 78.4 | 71.4 | 82.8 | 49.0 | 39.5 | 55.0 | 24.3 | 15.2 | 30.1 |
| Romania | 77.2 | 70.4 | 82.7 | 47.2 | 39.3 | 53.5 | 18.8 | 15.4 | 21.5 |
| Slovakia | 62.4 | 58.8 | 65.2 | 33.5 | 28.6 | 47.5 | 13.5 | 9.6 | 16.7 |

Supplementary Table 5 - P-values for differences between age groups regarding risk factors for COVID-19

|  | Hyperten-sion | Chronic lung disease | Cardiovas-cular disease | Cancer | Diabetes | Obesity | At least one risk factor | At least two risk factors | At least three risk factors |
| --- | --- | --- | --- | --- | --- | --- | --- | --- | --- |
| **50-59 (ref)** |  |  |  |  |  |  |  |  |  |
| 60-69 | <0.001 | 0.137 | <0.001 | 0.001 | <0.001 | <0.001 | <0.001 | <0.001 | <0.001 |
| 70-79 | <0.001 | <0.001 | <0.001 | <0.001 | <0.001 | 0.646 |  | <0.001 | <0.001 |
| 80-89 | <0.001 | <0.001 | <0.001 | <0.001 | <0.001 | 0.078 |  | <0.001 | <0.001 |
| 90+ | <0.001 | 0.005 | <0.001 | 0.005 | <0.001 | <0.001 |  | <0.001 | <0.001 |
| **60-69 (ref)** |  |  |  |  |  |  |  |  |  |
| 50-59 | <0.001 | 0.137 | <0.001 | 0.001 | <0.001 | <0.001 | <0.001 | <0.001 | <0.001 |
| 70-79 | <0.001 | <0.001 | <0.001 | <0.001 | <0.001 | <0.001 |  | <0.001 | <0.001 |
| 80-89 | <0.001 | <0.001 | <0.001 | 0.005 | <0.001 | <0.001 |  | <0.001 | <0.001 |
| 90+ | 0.013 | 0.030 | <0.001 | 0.199 | 0.013 | <0.001 |  | <0.001 | <0.001 |
| **70-79 (ref)** |  |  |  |  |  |  |  |  |  |
| 50-59 | <0.001 | <0.001 | <0.001 | <0.001 | <0.001 | 0.646 | <0.001 | <0.001 | <0.001 |
| 60-69 | <0.001 | <0.001 | <0.001 | <0.001 | <0.001 | <0.001 |  | <0.001 | <0.001 |
| 80-89 | 0.004 | 0.021 | <0.001 | 0.150 | 0.480 | 0.120 |  | <0.001 | <0.001 |
| 90+ | 0.022 | 0.573 | <0.001 | 0.598 | 0.519 | <0.001 |  | 0.097 | 0.067 |
| **80-89 (ref)** |  |  |  |  |  |  |  |  |  |
| 50-59 | <0.001 | <0.001 | <0.001 | <0.001 | <0.001 | 0.078 | <0.001 | <0.001 | <0.001 |
| 60-69 | <0.001 | <0.001 | <0.001 | 0.005 | <0.001 | <0.001 |  | <0.001 | <0.001 |
| 70-79 | 0.004 | 0.021 | <0.001 | 0.150 | 0.480 | 0.120 |  | <0.001 | <0.001 |
| 90+ | 0.001 | 0.621 | 0.554 | 0.930 | 0.358 | <0.001 |  | <0.001 | 0.001 |

Supplementary Table 6 - Prevalence (in percent) of having at least one, two and three risk factors for COVID-19 without including age as an underlying factor

|  | At least one risk factor | At least two risk factors | At least three risk factors |
| --- | --- | --- | --- |
| **All countries** |  |  |  |
| Total | 60.3 | 27.9 | 9.5 |
| Men | 61.9 | 28.8 | 10.1 |
| Women | 59.0 | 27.2 | 9.0 |
| **Northern EU** |  |  |  |
| Total | 56.7 | 25.1 | 8.0 |
| Men | 58.7 | 27.0 | 8.5 |
| Women | 54.9 | 23.5 | 7.6 |
| **Western EU** |  |  |  |
| Total | 59.1 | 27.6 | 9.5 |
| Men | 62.1 | 29.5 | 10.9 |
| Women | 56.4 | 26.0 | 8.3 |
| **Southern EU** |  |  |  |
| Total | 58.8 | 24.9 | 8.0 |
| Men | 62.1 | 26.3 | 8.4 |
| Women | 56.0 | 23.8 | 7.5 |
| **Eastern EU** |  |  |  |
| Total | 65.6 | 33.2 | 12.0 |
| Men | 62.0 | 31.2 | 11.3 |
| Women | 68.4 | 34.8 | 12.6 |

Supplementary Table 7- Burden (raw numbers) of risk factors for Covid-19 by age groups

|  | Hyperten-sion | Chronic lung disease | Cardiovas-cular disease | Cancer | Diabetes | Obesity | At least one risk factor | At least two risk factors | At least three risk factors |
| --- | --- | --- | --- | --- | --- | --- | --- | --- | --- |
| **Age 50-59** |  |  |  |  |  |  |  |  |  |
| Total | 17,617,257 | 2,630,354 | 3,914,946 | 1,896,302 | 4,954,854 | 13,212,943 | 28,566,871 | 10,827,272 | 3,486,749 |
| Men | 9,659,613 | 1,599,873 | 2,686,580 | 754,657 | 2,897,884 | 6,882,474 | 15,545,939 | 6,067,444 | 2,113,040 |
| Women | 7,963,078 | 1,022,496 | 1,239,390 | 1,115,451 | 2,044,993 | 6,351,872 | 13,044,576 | 4,771,650 | 1,363,329 |
| **Age 60-69** |  |  |  |  |  |  |  |  |  |
| Total | 21,653,735 | 2,706,717 | 5,933,956 | 2,290,299 | 6,766,792 | 13,013,062 | 40,184,335 | 23,007,093 | 10,046,084 |
| Men | 10,386,362 | 1,258,205 | 3,577,250 | 986,828 | 3,675,933 | 6,167,673 | 19,317,153 | 11,373,189 | 5,057,492 |
| Women | 11,253,819 | 1,451,222 | 2,354,814 | 1,314,315 | 3,094,116 | 6,845,389 | 20,864,744 | 11,637,160 | 4,983,443 |
| **Age 70-79** |  |  |  |  |  |  |  |  |  |
| Total | 19,150,987 | 2,457,108 | 7,046,118 | 2,312,572 | 6,684,779 | 7,660,395 | 36,133,938 | 25,944,167 | 13,008,218 |
| Men | 8,035,435 | 1,150,201 | 3,658,279 | 1,230,076 | 3,290,854 | 3,019,279 | 15,975,019 | 11,645,789 | 5,894,782 |
| Women | 11,107,564 | 1,310,330 | 3,406,857 | 1,088,582 | 3,406,857 | 4,636,551 | 20,158,919 | 14,292,674 | 7,116,098 |
| **Age 80-89** |  |  |  |  |  |  |  |  |  |
| Total | 11,425,634 | 1,670,057 | 5,539,701 | 1,140,527 | 3,910,377 | 4,032,577 | 20,366,549 | 15,722,976 | 8,166,986 |
| Men | 3,942,681 | 776,358 | 2,283,406 | 586,074 | 1,408,100 | 1,301,542 | 7,611,354 | 5,845,520 | 2,983,651 |
| Women | 7,474,544 | 880,108 | 3,252,575 | 561,229 | 2,500,018 | 2,729,612 | 12,755,195 | 9,872,521 | 5,178,609 |
| **Age 90+** |  |  |  |  |  |  |  |  |  |
| Total | 1,900,448 | 300,704 | 1,142,674 | 228,535 | 693,623 | 348,816 | 4,009,383 | 2,734,399 | 1,270,974 |
| Men | 438,658 | 105,956 | 288,201 | 55,097 | 186,483 | 62,514 | 1,059,561 | 671,762 | 308,332 |
| Women | 1,471,961 | 191,738 | 855,448 | 176,989 | 504,420 | 294,982 | 2,949,822 | 2,070,775 | 967,542 |

Data was estimated by multiplying the prevalence of risk factors in the SHARE population (Table 2) with the population number (assessed on January 1, 2017) in the respective countries based on the Eurostat Database
